# A Rare Case of Brain Metastasis from Dedifferentiated Acinic Cell Carcinoma of the Parotid Gland and Review of the Literature

**DOI:** 10.30699/IJP.2021.530574.2645

**Published:** 2021-08-20

**Authors:** Elham Mirzaian, Shafighe Asgari Karchekani, Ashkan Abdoli

**Affiliations:** 1 Department of Pathology, Shariati Hospital, School of Medicine, Tehran University of Medical Sciences, Tehran, Iran

**Keywords:** Acinic Cell Carcinoma, Dedifferentiation, Metastasis, Neoplasm, Salivary gland

## Abstract

Acinic cell carcinoma (ACC) is a low or intermediate-grade malignant epithelial neoplasm of the salivary glands that generally shows an indolent behavior. Most cases arise in the major salivary glands, especially the parotid gland. ACC is usually known as a low-grade malignant tumor; however, poorly differentiated and high-grade transformed variants have been reported and may tend to be late recurrence and metastasis.

Our case was a 49-year-old woman with a history of a right parotid rapidly growing mass lesion, which was cytologically diagnosed as monomorphic adenoma on fine-needle aspiration (FNA). Finally, the diagnosis of ACC with dedifferentiated components was reported, followed by parotidectomy. After 30 sessions of radiotherapy, she presented with hemifacial paresis. An imaging examination of the brain showed intracranial hemorrhage, and she underwent a craniotomy. We performed histopathological and immunohistochemical (IHC) examinations and diagnosed metastatic ACC with dedifferentiated components.

Few ACC cases with dedifferentiated components and with aggressive behavior have been reported in the literature , and to the best of our knowledge, this article is the first English report in Iranian population.

## Introduction

Acinic cell carcinoma (ACC) is a rare malignant epithelial neoplasm of the salivary gland that occurs more frequently in the parotid gland. The incidence of ACC is slightly higher in women than in men, and the median incidence age is 52. Histologically, ACC shows multidirectional differentiation towards acinar, ductal as well as myoepithelial cells, although the acinar cell differentiation is the most characteristic element. Acinar cells are polygonal with lightly basophilic cytoplasm and diastase resistant periodic acid-Schiff (PAS-D) positive zymogen granules. The growth pattern may be predominantly solid, microcystic, papillary–cystic, or follicular (1-6). Radiation exposure and familial predisposition are possible risk factors for ACC (4, 7).

ACC is generally a low-grade malignant tumor; however, some variants with high-grade transformation or dedifferentiation are associated with late recurrence, metastasis, and aggressive behavior (6, 8, 9). The recurrence rate is about 35%, with a notable tendency for late recurrence, up to 30 years after the initial presentation. Metastases occur in about 12% of all ACCs, mainly through the hematogenous way. The most common sites are cervical lymph nodes, lungs, and bones. Other less common reported metastases are cavernous sinus, spine, sternum, orbit, liver, skin, and intracranial space (4, 6).

Stanley *et al. *reported the first case of dedifferentiated ACC from the parotid gland in 1988 (10). Since then, few cases of ACC with dedifferentiated components and aggressive behavior have been reported (11-13). This article reports a case of a parotid gland ACC with a dedifferentiated component and brain metastasis.

## Case Presentation

**Fig. 1:**
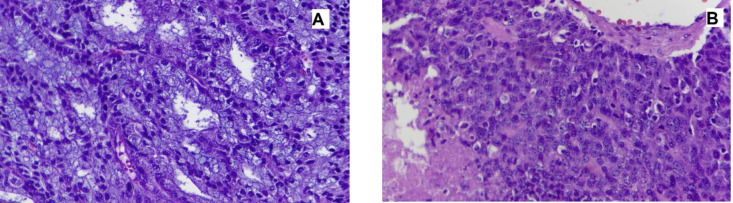
Histologic examination of parotid gland ma**ss**. A) A neoplasm is composed of different cell types arranged in follicular and microcystic patterns. Most tumor cells are large polygonal with a round to small oval nuclei, stippled chromatin and abundant basophilic granular cytoplasm (H&E stain, 400X). B) Dedifferentiated component with a solid pattern showing nuclear pleomorphism and increased mitotic rate with small foci of tumor necrosis (lower left) (H&E stain, 400X)

**Fig. 2:**
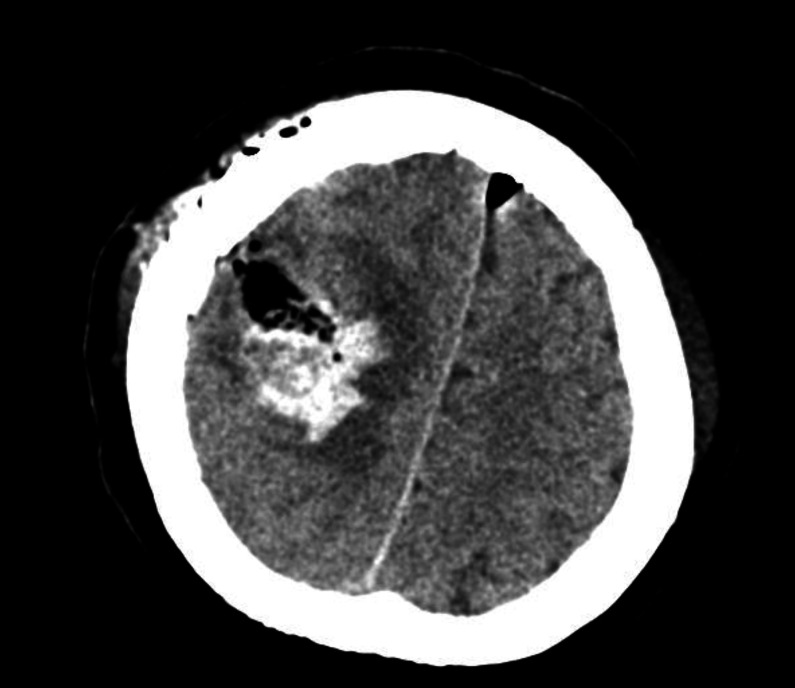
Brain CT scan. A heterogeneous intraparenchymal cerebral hemorrhage in the right frontoparietal lobe with surrounding edema

**Fig. 3:**
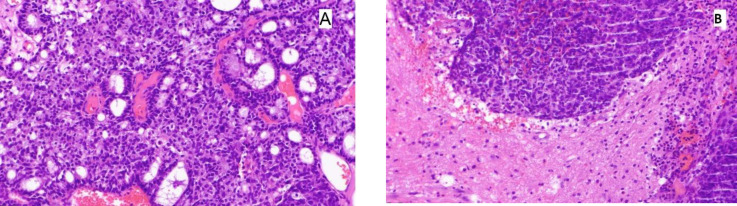
Histologic examination of the brain lesion. A) A neoplasm showing the same histomorphologic features described in the previous parotid gland mass (H&E stain, 200X), B) Brain parenchyma with tumor invasion (H&E stain, 200X)

**Fig. 4:**
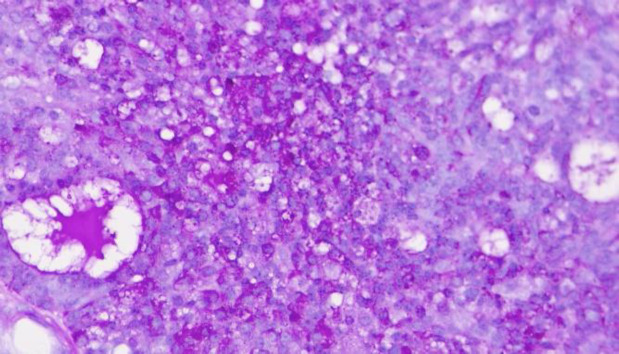
PAS-D staining. The cytoplasmic granules demonstrate PAS-D positivity (400X)

**Fig. 5:**
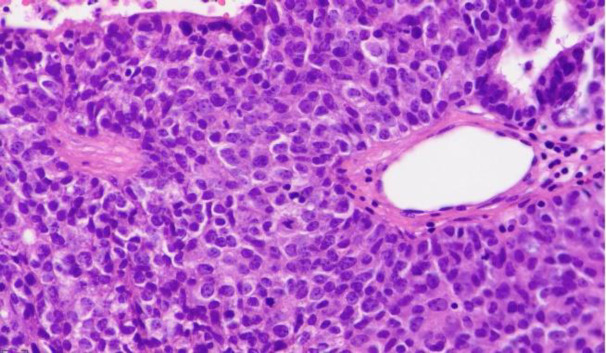
Histologic examination of the brain lesion. Foci of undifferentiated carcinoma with solid growth pattern showing nuclear pleomorphism and increased mitosis (H&E stain, 400X)

**Fig. 6:**
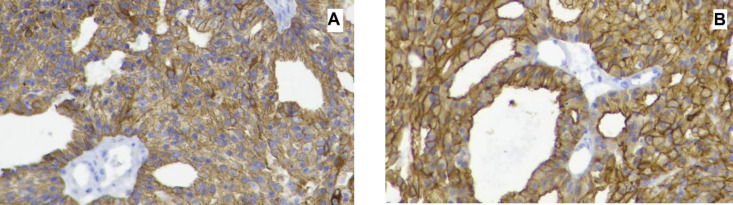
IHC stain. Tumor cells were positive for cytokeratin (A) and showed DOG 1 positivity with apical membranous staining (B), (IHC 400X)

Our case was a 49-year-old woman with a history of a right parotid painless mass lesion, which had grown 5-6 cm over eight months. Fine needle aspiration cytologic evaluation of parotid lesion was consistent with monomorphic adenoma, based on available cytologic reports from another center. The patient underwent a right parotidectomy at the same center. Histological examination indicated ACC with dedifferentiated components. Some adverse histologic factors, including more than two mitoses/10 HPFs, perineural/vascular invasion, tumor necrosis, and extracapsular invasion, were also identified on microscopic examination (Figure 1, A and B). The patient underwent t 30 sessions of radiotherapy. After two months, she experienced sudden severe left hemifacial paresis. Brain CT scan imaging demonstrated a 45*40 mm heterogeneous itrapa-renchymal cerebral hemorrhage in the right frontoparietal lobe with surrounding edema, which caused a 6 mm midline shift to the left side (Figure 2). She became a candidate for craniotomy at our center with the clinical impression of intracranial hemorrhage. Tissue samples were sent to the pathology department. Gross examination of the specimen showed multiple fragments of tanish gray soft fragile tissue. Microscopic study of the brain tissue showed a neoplasm composed of a mixture of different cell types, including most large polygonal cells with round to small oval nuclei, stippled chromatin pattern, and abundant basophilic granular cytoplasm. Some vacuolated cells and some cuboidal cells with eosinophilic cytoplasms and small dark nuclei were arranged in solid, follicular, and microcystic patterns (Figure 3 A and B). The cytoplasmic granules demonstrated diastase-resistant periodic acid-Schiff (PAS-D) positivity (Figure 4). Some foci were also observed with histologic features of the undifferentiated carcinoma, composed of atypical cells with pleomorphic nuclei, prominent nucleoli, and abundant mitotic figures (Figure 5). In the immunohistochemical study, tumor cells were positive for cytokeratin and showed DOG 1 positivity with apical membrane staining (Figure 6). Therefore, metastatic ACC with dedifferentiated components was diagnosed.

## Discussion

Salivary gland tumors originate from the major or minor salivary glands, either benign or malignant. Most tumors occur in the parotid gland, but their malignancy potential is less than submandibular, sublingual, and minor salivary glands tumors (14).

ACC constitutes 1-6% of malignant salivary gland tumors and mainly occurs in the parotid gland (81-98%), followed by the submandibular gland (11%) and minor salivary glands (3-12%) (2, 7). 

Patients with ACC typically present with a painless mass or swelling of the salivary glands (15). A solitary, encapsulated mass with a soft consistency and grey-white cut surfaces is usually seen on gross examination. Recurrent lesions are more commonly lobulated, may be unencapsulated, and show areas of necrosis. The acinar cell is the main histological differentiation of ACC. However, some other cell types, including intercalated ductal, vacuolated, clear, and non-specific glandular, are also recognized. The main growth patterns are solid-lobular, microcytic papillary-cystic, and follicular (4, 6, 15, 16). Complete surgical excision is the most effective treatment for acinic cell carcinoma. Postoperative radiation therapy may be helpful in cases with questionable margins, residual tumor after resection, and adverse pathologic characteristics, including a high tumor stage, neck node metastasis, high-grade transformation, perineural invasion, and lymphovascular invasion (17-19).

Most ACCs show a positive reaction for CK7 and CAM 5.2 in the IHC study. The tumor cells are commonly negative for p63 and CK20. DOG1 is expressed in normal serous acini and mucus acini (apical membranous staining pattern), and distal intercalated ducts (apical staining). Intense DOG1 expression, accompanied by the periodic acid– Schiff (PAS) reaction after diastase treatment, helps differentiate ACCs from morphologic mimics (20, 21).

Although ACC is overall a low-grade malignancy, it may show an unpredictable clinical course, especially regarding a subgroup of ACC described as “ACC with the dedifferentiated or high-grade transformation” (8, 9). Stanley *et al.* reported the first case of dedifferentiated ACC in 1988 (10). The high-grade transformation is also described in other salivary gland carcinomas such as adenoid cystic carcinoma and mucoepidermoid carcinoma (22, 23). The etiopathogenesis of the dedifferentiation and high-grade transformation is unknown (24). 

"Dedifferentiation" is defined as an abrupt transformation of a well-differentiated tumor into a high-grade morphology, which lacks the histologic features of the primary conventional tumor. The conventional and high-grade areas are sharply demarcated; however, a transitional zone can be identified in some cases (24). The high-grade component is characterized by atypical cells with pleomorphism, prominent necrosis, and high rate of cell proliferation (Ki67 index). Recent large studies around the development and progression of salivary gland tumors suggested some genetic changes could be related to ACC. The results revealed a deletion in tumor suppressor genes such as CDKN2A, B and chromosomal alternation on 4p, 5q, 6p, and 17p. These molecular changes were associated with tumor development. Also, deletions of chromosome 6q, loss of Y, and trisomy 21 have been reported in some ACCs (25, 26). Of note, Ska'lova *et al. *reported that TP53 gene mutation and HER2 amplification had not been detected in nine examined cases of dedifferentiated ACC. All nine cases showed intense membrane staining for β-catenin in the high-grade component vs. mildly cytoplasmic and nuclear staining in the low-grade areas. Cyclin-D1 expression and Ki67 index were higher in the high-grade component than in the low-grade foci (9). Recently, Lauren *et al.* also confirmed abnormal Cyclin-D1 and β-catenin IHC staining in high-grade transformed ACC (8).

There is no WHO histologic grading system for the classification of ACC. Gomez *et al. *suggested the "proliferative grading system", which recognizes high-grade neoplasms only based on high mitotic rate [>2 mitoses/10 HPFs] and presence of necrosis (27). These data are similar to older studies (28, 29) and confirmed by recent studies (17, 30).

The reported recurrence rate of ACC is about 35%, and it mostly happens late – some reports show tumor recurrence, even after 30 years from the initial presentation (31). Metastases occur in 0-30% of ACCs and tend to be hematogenous rather than lymphatic (32, 33). The most common sites are the cervical lymph nodes, lungs, and bones. Although not common, other reported sites for metastases are cavernous sinus, spine, sternum, orbit, liver, skin, and intracranial space (1, 4, 6, 34). Based on the most recent literature, the main prognostic factors that may predict poor outcome and metastasis include extracapsular extension, facial nerve involvement, positive surgical margins as well as high pathologic grade as evidenced by >2 mitoses/10 high power fields, cellular atypia, necrosis, perineural and vascular invasion and desmoplastic stromal reaction (35, 36). Of note, Fang *et al.* suggested that high-grade transformation is an independent predictor factor of distant metastases in parotid ACC (37). Khelfa *et al.* reported a 78-year-old female with a history of right parotid ACC, which presented with pelvic metastasis one year after diagnosis (6). Vidyadhara *et al.* also reported a 40-year-old man presented with sudden-onset severe back pain and neurological signs such as tingling, numbness, and spasticity of both lower limbs. The patient had a previous history of incomplete parotid gland resection and was diagnosed with ACC through histological examination. Magnetic resonance imaging (MRI)of the spine revealed a mass lesion of the T4 vertebra. He underwent posterior decompressive laminectomy of T4 vertebra, and the Histopathological examination was conclusive of metastatic ACC (1). Al-Otaibi *et al.* reported a 48-year-old woman with histological diagnosis of ACC of the parotid gland with high-grade transformation, perineural invasion, positive surgical margins, and regional lymph node involvement. Less than a year later, the patient developed widespread distant metastases at multiple sites, including the brain, lungs, liver, bones, and retroperitoneum (38). Most of the reported cases were over 40 years old, similar to our case. In most patients, metastases occurred after 6-12 months from initial parotidectomy, while in our case, they occurred within two months. Most of the reported metastases were to the bones, lungs, and brain. Unfavorable factors such as extensive local invasion, positive surgical margins, lymph node involvement, and high-grade transformation were observed in the histologic examination of most reported parotid gland ACCs. 

About our case, although histologic features were suggestive of poor prognosis, lymph node dissection while parotidectomy and IHC study for ki67 was not performed. Therefore, exact staging of the initial ACC of the parotid gland was not possible.

The reported prognosis and survival data are generally favorable, with a 10-year overall survival rate of >80% (27, 39), but Metastatic ACC has a poor prognosis with 5-year survival rates of less than 50% (40). Therefore, ACC should be considered a malignancy with an unpredictable clinical course due to its propensity to recur, metastasize and even lead to death, especially a subgroup described as "ACC with dedifferentiation or high-grade transformation" (41). Due to notably high tendency of latent recurrence and metastasis more than 20 or 30 years after initial treatment in ACC cases, long-term patient follow-up is mandatory.

## Conclusion

We reported a rare case of brain metastasis of a parotid dedifferentiated ACC primary. Although ACCs are infrequently accompanying metastasis, in a patient with a history of ACC presented with new unexplained signs and symptoms, a metastatic lesion must be kept in mind as an important differential diagnosis. Therefore, long-term follow-up of the patient is recommended.

## Conflict of Interest

The authors declared no conflict of interest. 
